# Effects of diets supplemented by fish oil on sex ratio of pups in bitch 

**Published:** 2016-06-15

**Authors:** Faramarz Gharagozlou, Reza Youssefi, Vahid Akbarinejad

**Affiliations:** 1 *Department of Theriogenology, Faculty of Veterinary Medicine, University of Tehran, Tehran, Iran; *; 2 *Young Researchers and Elites Club, Roudehen Branch, Islamic Azad University, Roudehen, Iran.*

**Keywords:** Dog, n-3 fatty acids, Secondary sex ratio

## Abstract

The present study was conducted to evaluate the effect of fish oil supplementation prior to mating on secondary sex ratio of pups (the proportion of males at birth) in bitches. Sixty five bitches (German Shepherd, n = 35; Husky, n = 30) were enrolled in the study. Bitches (140-150 days post-estrus) were given 2% per dry matter intake palm oil and fish oil in the control (n = 33) and treatment (n = 32) groups, respectively. To induce estrus, bitches were received equine chorionic gonadotropin (eCG) administration (50 IU kg^-1^) 30 days after nutritional supplementation followed by human chorionic gonadotropin (hCG) administration (500 IU per dog) seven days later. Bitches were introduced to dogs of the same breed after hCG administration. The weight of bitches was increased over time (*p* < 0.05), but their weight change was not different between two groups (*p* > 0.05). The mating rate, pregnancy rate and litter size were not influenced by treatment and breed. Secondary sex ratio was higher in the treatment (105/164; 64.00%) than in the control (68/147; 46.30%) group (*p* < 0.05; adjusted odds ratio = 2.068). Moreover, secondary sex ratio was higher in Husky bitches (88/141; 62.40%) compared to German Shepherd (85/170; 50.00%; *p* < 0.05; adjusted odds ratio = 1.661). In conclusion, the present study showed that inclusion of fish oil in the diet of bitches prior to mating could increase the proportion of male pups at birth. In addition, it appears that there might be variation among dog breeds with regard to the sex ratio of offspring.

## Introduction

It is generally expected that the proportion of sexes are equal at birth, however, several factors have been reported to skew the sex ratio of the offspring (the proportion of males) toward one gender.^1^^,^^2^ In this regard, Trivers and Willard hypothesized that mothers with greater body condition would be more likely to give birth to sons than daughters.^3^ This hypothesis was further substantiated by different studies in various species.^1^^,^^2^ The effect of maternal body condition on sex ratio of offspring has been associated with the level of maternal nutrition.^1^ Food restriction prior to conception is known to decrease the proportion of male pups in the litters of mice^4^^,^^5^ and rats.^6^ The effect of maternal nutrition on sex ratio has been attributed to maternal glucose concentration since *in vitro* studies has revealed sexual dimorphism of embryos in response to glucose during the early stages of embryo-genesis.^7^^,^^8^ The presence of glucose in the culture medium detrimentally impacts the development of female embryos and inhibits their transition from morula to blastocyst stage,^9^^,^^10^ consequently leading to faster development of male embryos, and in turn, male-biased sex ratio.^9^^-^^12^

Nevertheless, it has been shown that the effect of maternal nutrition is not merely through alteration of body condition with the composition of the maternal diet playing a significant role in sex ratio adjustment as well.^1^ Rosenfeld *et al*. observed an effect of dietary fat content in mice with mothers fed on a low fat diet having a female-biased sex ratio while those fed on a very high fat diet had a male-biased sex ratio;. This effect was not associated with mother’s body mass.^13^ Moreover, the fatty acid composition of diet has been observed to influence sex ratio in mice. A diet enriched with n-3 fatty acids had no effect on the sex ratio of pups, while a diet enriched with n-6 fatty acids skewed the sex ratio of pups toward females.^14^ By contrast, consumption of diet fortified with n-6 fatty acids resulted in a male-biased sex ratio of embryos in sheep.^15^

Poly unsaturated fatty acids (PUFAs) are fatty acids that possess more than one carbon-carbon double bond in their *chain*. The PUFAs consist of different series of fatty acids including n-3 and n-6 ones.^16^ Linoleic acid (C18:2) is the predominant dietary n-6 PUFA, which would be further converted to arachidonic acid (C20:4) in the body. Arachidonic acid is the precursor of various inflammatory mediators including prostaglandins, prostacyclin, throm-boxanes and leukotrienes.^16^ The main dietary sources of linoleic acid are vegetable oils including corn, soybean, sunflower and safflower oils.^16^ On the other hand, linolenic acid (C18:3), eicosapentaenoic acid (C20:5) and docosa-hexaenoic acid (C22:6) are the predominant dietary n-3 PUFAs,^16^ which have anti-inflammatory properties, acting through inhibition of the formation of n-6 PUFAs’ derivatives.^17^^,^^18^ The main dietary source of linolenic acid is linseed oil, and the main source of eicosapentaenoic and docosahexaenoic acids is fish oil.^16^

In a kennel located in Pardis, Tehran, Iran, the recorded secondary sex ratio of pups (the proportion of male pups at birth) was 47.50% (113/238) from 2009 to 2010, which was quite comparable with the values of sex ratio in two populations of domestic dogs (50.50 % and 49.60%) reported by Gavrilovic *et al*. in Sweden. Fish oil had been added to the diet of bitches for a 4-month period in 2011, which would further resulted in a secondary sex ratio of 61.20% (30/49) in bitches.^19^ Based on this observation, we hypothesized that fish oil supplementation might increase the proportion of male pups in bitch. The present study was conducted to evaluate the effect of fish oil compared to palm oil, a source of saturated and monounsaturated fatty acids, on secondary sex ratio of offspring in bitches.

## Materials and Methods

**Animals. **The Animal Care Committee of Faculty of Veterinary Medicine, University of Tehran, Tehran, Iran, approved the present study in terms of animal welfare and ethics. Sixty five healthy bitches (German Shepherd, n = 35; Husky, n = 30), aged 3 to 5 years and weighing 19.80 to 34.20 kg were enrolled in the study from December 2012 to June 2013. All bitches were housed in a kennel located in Damavand, Tehran, Iran. Bitches were confirmed to be in anestrus using serum progesterone concentration (< 1 ng mL^-1^) and vaginal cytology. Day of anestrus was determined based on the reproductive records of each individual animal.

**Fig. 1:**
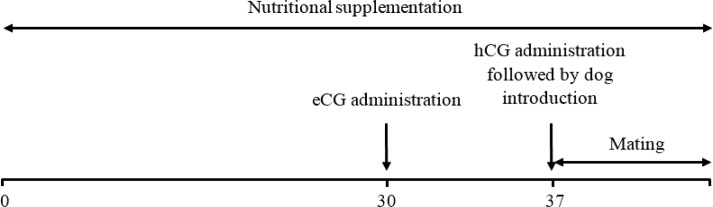
Experimental design of the study. Bitches were supplemented with palm and fish oils from day 0 (140 to 150 days post-estrus) to mating. Estrus induction was implemented by administration of eCG and hCG on days 30 and 37, respectively. Bitches were introduced to dogs immediately after hCG administration

**Experimental design. **Bitches were randomly assigned to two experimental groups by breed 140 to 150 days post-estrus (Day 0). All bitches were fed on a commercial dry dog food (Bosch PetFood GmbH & Co. KG, Blaufelden, Germany) as the manufacturer indicated and supplied with water *ad libitum*. Additionally, from day 0 until mating, bitches in the control group (German Shepherd, n = 18; Husky, n = 15) received palm oil (Table 1) at 2.00% of dry matter intake while bitches in the treatment group (German Shepherd, n = 17; Husky, n = 15) received fish oil (Table 1) at 2.00% of dry matter intake. Thirty days after the commencement of nutritional supplementation, estrus was induced by intramuscular administration of 50 IU kg^-1^ equine chorionic gonadotropin (eCG; Intervet, Boxmeer, The Netherlands) followed by administration of human chorionic gonadotropin (hCG) seven days later (500 IU per dog; LG Life Sciences, Seoul, South Korea).^20^ Following hCG administration, bitches were introduced to dogs of the same breed with proven fertility (German Shepherd, n =3; Husky, n =3; Fig. 1). Mating was monitored using closed-circuit cameras. Pregnancy diagnosis was implemented using ultrasonography (Model Titan; SonoSite, Washington DC, USA) 30 days after mating. The weight of bitches was recorded at the beginning of the study and at the time of hCG administration. 

**Table 1:** Fatty acid composition of the palm and fish oil fed to bitches in the present study

**Fatty acid**	**Palm oil (%)**	**Fish oil (%)**
**Myristic acid (C14:0)**	1.10	2.40
**Palmitic acid (C16:0)**	40.10	21.30
**Palmitoleic acid (C16:1)**	0.40	5.20
**Stearic acid (C18:0)**	15.00	3.70
**Oleic acid (C18:1)**	32.50	28.20
**Linoleic acid (C18:2)**	6.80	7.60
**Linolenic acid (C18:3)**	0.30	2.00
**Eicosapentaenoic acid (C20:5)**	―	6.70
**Docosahexaenoic acid (C22:6)**	―	15.20
**Others**	3.80	7.70

**Reproductive parameters. **Mating rate was defined as the number of bitches that mated divided by the number of bitches assigned to the study. Pregnancy rate was defined as the number of bitches diagnosed pregnant divided by the number of bitches that mated. Secondary sex ratio was defined as the number of male pups divided by the number of all pups born.

**Statistical analysis. **Data associated with the weight of bitches before and after nutritional supplementation were analyzed using mixed procedure including random and repeated statements in the model to specify covariation between and within bitches, respectively.^21^ Data associated with litter size were analyzed using GLM procedure. Binary outcome variables including mating rate, pregnancy rate and secondary sex ratio were analyzed by multivariable logistic regression analysis using GENMOD procedure including function link logit in the model, in which breed and treatment were considered as fixed effects. The logistic regression analyses generated adjusted odds ratios (AORs) and 95.00% confidence intervals (CIs). Differences were considered significant at *p* < 0.05. All analyses were conducted in SAS (version 9.2, SAS Institute Inc., Cary, USA).

## Results

**Fig. 2:**
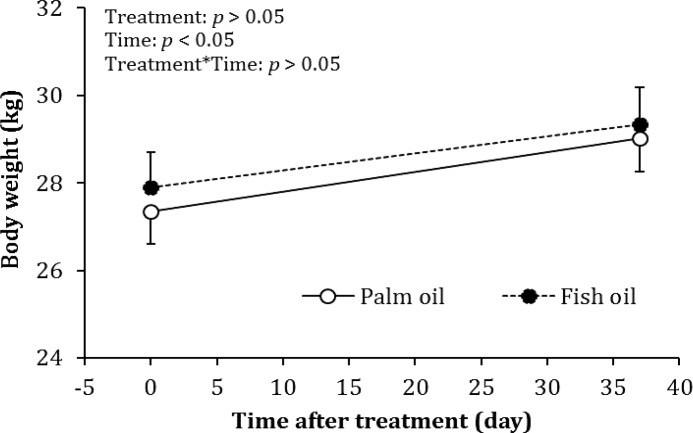
Body weight of bitches before and after nutritional supplementation in the control (palm oil) and treatment (fish oil) groups. Data are presented as mean ± SEM

At the beginning of the study, the weight of bitches was 27.36 ± 0.75 kg and 27.90 ± 0.81 kg in the control and treatment groups, respectively. At the time of hCG administration, the weight of bitches was 29.03 ± 0.76 kg and 29.33 ± 0.84 kg in the control and treatment groups, respectively. The weight of bitches did not differ between two experimental groups either at the beginning of the study or at the time of hCG administration (*p* > 0.05). But the weight of bitches was increased over time in response to nutritional supplementation (*p* < 0.05). Moreover, the interaction of treatment by time had no effect on the weight of bitches (*p* > 0.05; Fig. 2). 

Neither treatment nor breed influenced mating rate, pregnancy rate and litter size (*p* > 0.05; Table 2). Secondary sex ratio was higher in the bitches supplemented with fish oil (105/164 = 64.00%) than those supplemented with palm oil (68/147 = 46.30%; adjusted odds ratio = 2.06; *p* < 0.05; Tables 2 and 3). In addition, secondary sex ratio was higher in Husky (88/141 = 62.40%) than in German Shepherd (85/170 = 50.00%) bitches (adjusted odds ratio = 1.66; *p* < 0.05; Tables 2 and 3).

**Table 2:** Reproductive performance of bitches in the control (palm oil) and treatment (fish oil) groups considering breed. Data are presented as percentages and mean ± SEM. Numbers in parentheses are actual numbers

**Breed **	**Control**					**Treatment**			
	**MR**	**PR**	**LS**	**SSR**		**MR**	**PR**	**LS**	**SSR**
**Husky**	93.30(14/15)	71.40(10/14)	6.00 ± 0.50	53.80(35/65)		93.30 (14/15)	92.90(13/14)	5.90 ± 0.40	69.70(53/76)
**German Shepherd**	88.90(16/18)	81.30(13/16)	6.30 ± 0.60	40.20(33/82)		100.00 (17/17)	82.30(14/17)	6.30 ± 0.60	59.10(52/88)

**MR**: Mating rate, **PR**: Pregnancy rate, **LS**: Litter size, and **SSR**: Secondary sex ratio.

**Table 3:** Effects of treatment and breed on secondary sex ratio (SSR) in Husky and German Shepherd bitches fed on fish and palm oil at the level of 2.00 % of dry matter intake prior to mating

**Effect**	**Class**	**SSR (%)**	**Estimate ± SE**	**AOR**	**95% CI**	***p*** **-value**
**Treatment**	Fish oil	64.00 (105/164)	0.50 ± 0.23	2.06	1.30 - 3.27	*p* < 0.05
	Palm oil	46.30 (68/147)	―	―	―	―
**Breed**	Husky	62.40 (88/141)	0.72 ± 0.23	1.66	1.04 - 2.63	*p* < 0.05
	German Shepherd	50.00 (85/170)	―	―	―	―

**SE**: Standard error, **AOR**: Adjusted odds ratio, and **CI**: Confidence interval.

## Discussion

The present study revealed that inclusion of fish oil (a source of n-3 fatty acids) could skew secondary sex ratio of offspring toward male pups in dogs. By contrast, feeding n-3 fatty acids has been reported to have no effect on the sex ratio of offspring in mice^14^ and sheep.^22^ As a result, it could be speculated that the effect of n-3 fatty acids on sex ratio might be species-specific. In this regard, species-specific effects of n-6 fatty acids have been reported previously. Fountain *et al*. found female-biased sex ratio in mice following supplementation with diet enriched with n-6 fatty acids,^14^ whereas Green *et al*. reported male-biased sex ratio in ewes fed a diet enriched with n-6 fatty acids.^15^ Indeed, the oocyte experiences peculiar phenomena during the pre- and post-ovulatory period in canine compared to other mammalian species,^23^ including early acquisition of luteinizing hormone (LH) receptors by follicles,^24^ ovulation of immature oocytes,^25^ pre-ovulatory luteinization of follicular wall and the consequent rise in progesterone concentration,^26^ and prolonged period of time required for the oocyte to remain within oviduct for maturation after ovulation and before fertilization.^23^ In addition, the female genital tract, i.e. uterus and uterotubal junction, serves as a reservoir for sperm following semen deposition, during which the interaction between female genital tract and sperm helps the spermatozoa have prolonged longevity until fertilization.^27^^,^^28^ Taken together, the specific characteristics of canine reproductive physiology influence the development of not only the oocytes but also the embryos^23^ and might contribute to the specific effect of n-3 fatty acids on sex ratio in dogs compared to other species.

Given that the weight of bitches was not different between treatments either before or after fat supplementation, the effect of fish oil on sex ratio of pups was unlikely to be attributable to any change of bitches’ body mass in the present study. 

Rosenfeld *et al*. observed male-biased sex ratio of pups in mice fed on a diet containing a very high fat versus a low fat diet level.^13^ A high fat diet can increase concentrations of circulating estradiol when compared to low fat diet.^29^ Investigating the impact of estradiol on *in vitro* produced embryos in mice, Zhang *et al*. reported that high concentrations of estradiol in the culture medium resulted in a male-biased sex ratio.^30^ More recently, administration of estradiol prior to insemination has been observed to augment the probability of male calves being born in cattle.^31^ Women receiving fish oil have been found to have higher circulatory estrogens than those received thistle oil, which contains very limited amount of n-3 fatty acids.^32^ Hence, it could be concluded that a potentially higher circulating estrogen concentration with fish oil versus palm oil supplementation could have been contributed to male-biased sex ratio in the present study. Interestingly, the effect of estradiol on sex ratio of offspring has been observed to be species-specific as was the effect of n-3 fatty acids on sex ratio of offspring.^30^^,^^31^^,^^33^ Nevertheless, circulatory estrogens of bitches were not measured in the present study. It remains to be elucidated by further studies.

Introducing Y- and X-bearing spermatozoa into the oviducts of the same sows, Almiñana *et al*. observed sex-specific transcriptomic responses, corroborating the contribution of oviduct in the sex allocation of offspring.^34^ Further organization of differentially expressed trans-criptomes revealed up-regulation of genes involved in immune system in response to Y than X sperms.^34^ Consumption of n-3 fatty acids inhibit the formation of arachidonic acid-derived eicosanoids,^17^^,^^18^ playing a central role in different aspects of immunity including cytokine production, antibody formation, differentiation, cell proliferation, migration and antigen presentation.^35^

Accordingly, the effect of fish oil on sex ratio of pups in the present study could alternatively be attributed to the effect of n-3 fatty acids on immune system-related signals of the oviduct and their impact on Y and X sperms.

Prostaglandin F_2α_ (PGF_2α_) regulates the oviductal contractions.^36^^,^^37^ Oviductal contractions facilitate the transport of both sperms and oocytes toward the site of fertilization, thereby influencing the time at which sperm and oocyte meet for fertilization.^23^^,^^28^^,^^37^ Time of fertilization relative to ovulation has been indicated to impact the sex of offspring in a wide range of species including bovine,^38^^-^^40^ deer,^41^ human,^42^^,^^43^ rabbit^44^ and rodents.^45^^,^^46^ As mentioned above, n-3 fatty acids could decrease production of arachidonic acid-derived eicosanoids including PGF_2α_.^17^^,^^18^ Therefore, alteration of oviductal contractility through inhibition of PGF_2α_ production might be another mechanism whereby feeding bitches with fish oil altered sex ratio in the present study.

In addition, the present study revealed the effect of breed on sex ratio of offspring. The effect of the breed of sire has been indicated in bovine^47^^,^^48^ and equine.^49^ To the best knowledge of the authors, the effect of the breed of dam on sex ratio of offspring has not been reported, however, the variation in sex ratio between German Shepherd and Husky breeds originated from the sire and/or dam could not be elucidated by the present study, because the present study was not primarily designed to assess the specific effect of breed of either sire or dam on sex ratio of pups, and it requires further studies with appropriate study design to be explained. 

In conclusion, the present study showed that inclusion of fish oil in the diet of bitches prior to mating could increase the proportion of male pups. This effect might be resulted from elevation of maternal estrogen concentrations, modulation of immune system-related signals of the oviduct and/or alteration of oviductal contractility. Further studies are required to unravel the mechanisms responsible for the effect of fish oil on sex ratio of offspring in bitch. Nevertheless, given that male pups are more desired in guard breeds of dogs, fish oil could be used to address the intention of breeders in this regard. In addition, it seems that there is variation among breeds of dogs in secondary sex ratio of offspring. Further studies investigating the effect of breed of dog on sex ratio could help identify the mechanisms underlying sex ratio adjustment in canine.
